# A Quality by Design Approach in Pharmaceutical Development of Non-Viral Vectors with a Focus on miRNA

**DOI:** 10.3390/pharmaceutics14071482

**Published:** 2022-07-16

**Authors:** Ioana Toma, Alina Silvia Porfire, Lucia Ruxandra Tefas, Ioana Berindan-Neagoe, Ioan Tomuță

**Affiliations:** 1 Department of Pharmaceutical Technology and Biopharmacy, Iuliu Hatieganu University of Medicine and Pharmacy, 400012 Cluj-Napoca, Romania; ioana0612@gmail.com (I.T.); tefas.lucia@umfcluj.ro (L.R.T.); tomutaioan@umfcluj.ro (I.T.); 2 Research Center for Functional Genomics Biomedicine and Translational Medicine, Iuliu Hatieganu University of Medicine and Pharmacy, 400337 Cluj-Napoca, Romania; ioana.neagoe@umfcluj.ro

**Keywords:** non-viral vectors, cancer, gene therapy, quality by design

## Abstract

Cancer is the leading cause of death worldwide. Tumors consist of heterogeneous cell populations that have different biological properties. While conventional cancer therapy such as chemotherapy, radiotherapy, and surgery does not target cancer cells specifically, gene therapy is attracting increasing attention as an alternative capable of overcoming these limitations. With the advent of gene therapy, there is increasing interest in developing non-viral vectors for genetic material delivery in cancer therapy. Nanosystems, both organic and inorganic, are the most common non-viral vectors used in gene therapy. The most used organic vectors are polymeric and lipid-based delivery systems. These nanostructures are designed to bind and protect the genetic material, leading to high efficiency, prolonged gene expression, and low toxicity. Quality by Design (QbD) is a step-by-step approach that investigates all the factors that may affect the quality of the final product, leading to efficient pharmaceutical development. This paper aims to provide a new perspective regarding the use of the QbD approach for improving the quality of non-viral vectors for genetic material delivery and their application in cancer therapy.

## 1. Introduction

Gene therapy represents the transfer of genetic material, a functional gene, or a DNA/RNA fragment into a specific cell [1]. ICH guideline S12 defines gene therapy products as “Therapeutic products that mediate their effect by the expression of transferred genetic materials, or by specifically altering the target genome of human cells.” [2]. According to the Food and Drug Administration (FDA), “Human gene therapy seeks to modify or manipulate the expression of a gene or to alter the biological properties of living cells for therapeutic use.” [3].

The crucial step in gene therapy is choosing the appropriate vector for specific gene delivery into the target cell [4]. The vector that delivers the genetic material is the most critical factor for successful gene therapy. Generally, vectors are classified into viral and non-viral groups. Viral vectors trigger immune responses that inhibit cargo delivery. Therefore, non-viral vectors have drawn significant attention over viral ones. This has resulted in an increased number of clinical trials using non-viral vectors, while the number of viral vector trials has decreased significantly [4,5]. Non-viral vectors have no risk of infection compared to viral ones [1]. Liposomes with cationic lipids are considered valuable non-viral delivery systems [6]. The first report on a cationic lipid-based system that proved successful in in vitro transfection studies dates back to 1987 [1]. The term “transfection” refers to the non-viral transfer of genetic material, which is based on cellular transport systems for uptake and expression in cells [7]. An important aspect of successful non-viral transfer of nucleic acid is their localization in the cytoplasm [8]. Multiple studies have demonstrated that gene therapy is an effective alternative in changing the expression of any gene of interest [9]. In this context, the development of new approaches for the diagnosis and treatment of aberrant miRNA expression in different diseases represents an important impulse for research [6]. The major issues with gene therapy development are preventing genotoxicity and immune responses that limit the in vivo administration of vectors, and also improving gene transfer for target diseases [10].

Over the past years, great efforts have been made toward developing these therapies as part of the standard treatment for human diseases. As expected, gene therapy provided durable benefits to human health, exemplified by scientific advances and clinical success [10]. Most gene therapy clinical trials have targeted cancer, inherited monogenic diseases (achieving the greatest successes in gene therapy to date), and cardiovascular disease. The number of trials entering late phases are constantly increasing [11]. Since cancer is one of the most distressing and life-threatening diseases, treating it requires the development of alternative strategies. Cancer is characterized by uncontrolled or inappropriate cell growth, leading to high mortality rates worldwide. Conventional cancer treatments include surgery, radiation, and chemotherapy, used either alone or in combination depending upon the stage and/or severity of the tumor metastasis. The conventional cancer treatment slightly improves the survival rate of patients, and the side effects are often severe. Moreover, the non-specific biodistribution of conventional cancer therapies limits their usage [12,13].

The Quality by Design (QbD) approach provides support for product and process understanding. The main goal of the QbD concept is to achieve product quality and robustness, to reduce product variability, and to increase pharmaceutical development and manufacturing efficiencies through sound and systematic analysis [14].

The novelty of the present paper is related to the two concepts, the non-viral vectors and the QbD approach. The paper emphasizes that implementing the QbD concept in nanotechnology-based genetic material delivery could bring a lot of advantages that are essential for large-scale production.

## 2. The Genetic Material Used in Cancer Gene Therapy

DNA and RNA are long, linear polymers consisting of a large number of linked nucleotides, each nucleotide being composed of a sugar, a phosphate, and a base. For the DNA structure, the bases form specific pairs which are stabilized by hydrogen bonds, resulting in a double helix formation. Both macromolecules carry genetic information stored in the sequence of bases along a nucleic acid chain. The genetic information lying in these base pairs is copied when a new chain is synthesized [12].

The classical DNA double helix defines a structural basis for the genetic code via defined base-pairing [15]. Typically, DNA exists in the genome of both prokaryotic and eukaryotic organisms in the double-stranded form [16]. Moreover, it is well known that DNA is a structurally dynamic and flexible molecule, adopting alternative structures such as single-stranded, three-stranded, and four-stranded variations [15,16]. A total of 98% of human DNA is represented by non-protein-coding sequences [17]. Following the DNA discovery, a long and intensive study has been performed to evidence the influence of RNA molecules in biology [18].

RNA synthesis is the process of transcribing DNA nucleotide sequence information into RNA sequence information by RNA polymerases. This process of transcription is followed by translation, the synthesis of proteins. Messenger RNA (mRNA) is a heterogeneous class of molecules and represents the template for protein synthesis, being the information-carrying intermediate in protein synthesis. Transfer RNA (tRNA) carries activated amino acids to the ribosome for peptide bond formation in a sequence dictated by the mRNA template. Ribosomal RNA (rRNA) plays a catalytic and a structural role in protein synthesis, since it is the major component of ribosomes [12].

Due to the high thermodynamic stability and amazing diversity of RNA in terms of structure and function, its encapsulation in nanocarriers is very promising compared to that of DNA [6]. RNA is structurally similar to DNA, but the chemical composition and functions are substantially different [19]. Based on their protein-coding potential, RNAs are classified as coding RNAs (cRNAs) that usually refer to mRNA, and non-coding RNAs (ncRNAs) [20].

**Figure 1:**
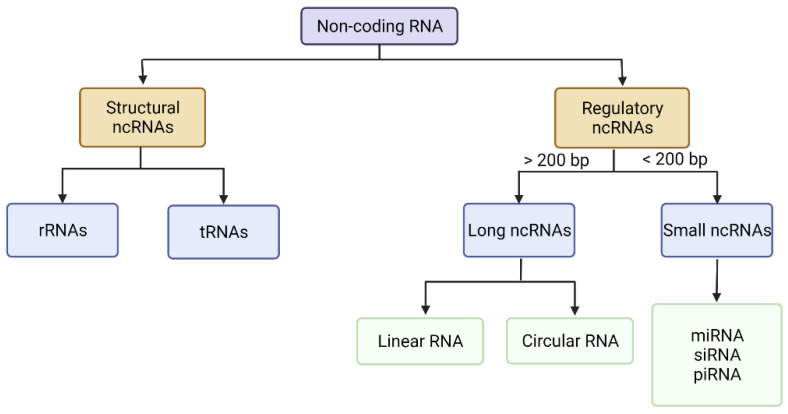
ncRNAs classification [19,21]. (Figure was created with BioRender.com, (accessed on 13 June 2022)). Note: Adapted from [19,21].

The human genome is mostly comprised of non-coding DNA, which is transcribed into different categories of ncRNAs [21]. Less than 3% of the genome is translated into coding transcripts [20,21]. NcRNAs are RNA species that can be classified into structural ncRNAs, which contain rRNAs and tRNAs, and regulatory ncRNAs [21,22]. NcRNAs can be further classified by length (the number of base pairs, bp) as either small ncRNAs of 18–200 bp or long ncRNAs of more than 200 bp (Figure 1) [17]. NcRNAs participate at the transcriptional and post-transcriptional level in regulating gene expression or cellular processes and pathways in developmental and pathological conditions [21,23]. NcRNAs exert their functions in gene regulation and cell signalling networks without encoding proteins [20,23]. Studies have demonstrated the implication of ncRNAs in competitive regulatory interactions that often cause modifications in complex regulatory systems, leading to malignancies. All lncRNAs classes share a common functionality in their ability to modulate gene expression, and abnormalities in their expression has been implicated in various diseases, including cancer development and progression [22].

### 2.1. Long Non-Coding RNAs (lncRNAs)

LncRNAs represent the largest class of ncRNAs from the mammalian genome [22]. LncRNAs are transcripts with more than 200 nucleotides transcribed by RNA polymerase II, but cannot be translated into proteins [21,22]. They exhibit various modes of action. Particularly, their gene expression regulatory function can be exerted at different levels, including epigenetic, transcriptional, post-transcriptional, and translational levels [20]. Physiological processes such as cell proliferation, differentiation, and apoptosis may be altered by lncRNAs that affect the regulatory mechanism of mRNA transcription, alternative splicing, and translation [21]. LncRNAs bind to proteins and can modulate enzyme activity [20]. Additionally, they can attach to other RNA molecules and regulate their translation [20,23].

### 2.2. Small Non-Coding RNAs

Small non-coding RNAs (sncRNAs) are divided into three major families for eukaryotic cells: micro-RNAs (miRNAs), PIWI-interacting RNAs (piRNAs), and small-interfering RNAs (siRNAs). These families have different origins, but they possess similitudes in their biosynthetic pathways and regulatory mechanisms [24]. SncRNAs act as multi-target inhibitors and activators with mRNAs’ 5′ untranslated region [25]. SncRNAs are regulators of genomic output at the transcriptional or post-transcriptional levels. The control of the regulatory process requires two groups of proteins: processors and effectors. The enzymes with nuclease activity, called processors, can cut small RNAs (sRNAs) from specific transcripts. The effectors are a group of RNA binding proteins that stabilize, transport, and regulate the activity of the sncRNAs [24].

#### 2.2.1. miRNA

MicroRNAs (miRNAs) are 18–23 nucleotide long ncRNA molecules that play a crucial role in regulating gene expression [6]. MiRNAs execute their post-transcriptional regulatory effects by binding to specific sites on their target transcripts, resulting in transcript degradation or translational inhibition [22]. Their profile is constantly modified in different stages of the disease, making miRNAs an excellent tool for targeted therapy through nanoparticles [6]. MiRNAs are involved in many cellular processes such as cell cycle, differentiation, proliferation, apoptosis, inflammation, stress response, energy metabolism, and immune response [26,27].

MiRNAs can activate or suppress gene expression [6]. Multiple miRNAs can regulate one gene, and a single miRNA can regulate the expression level of multiple genes and proteins [6,27]. Within a single cancer type, miRNA can be either oncogenic or tumor suppressive [28]. Oncogenes (OG) are overexpressed mutated genes that stimulate the growth of cancer cells. Tumor suppressor genes (TSG) or anti-oncogenes prevent tumor growth and are usually under-expressed in cancer cells [27]. In tumors, miRNAs can behave either as OG by targeting TSG, resulting in the development of cancer, or as TSG by targeting OG, which results in the formation of normal cells [27,29].

MiRNAs have demonstrated relevant application in research and pharmaceutical fields [6]. MiRNAs regulate gene expression by suppressing mRNA translation and, consequently, reducing the stability of mRNA [28]. MiRNAs are exported outside the nucleus, in the cytoplasm, where they are cleaved to mature miRNAs [30]. MiRNAs can also modulate the malignant transformation of cells, being involved in all cellular processes. They can differentiate the numerous subtypes of one particular cancer or identify specific oncogenic abnormalities, and are therefore considered valuable biomarkers in multiple diseases [29].

MiRNA-based therapies have two targets: tumor suppressor miRNAs that are downregulated, in which case it is necessary to restore their expression using synthetic miRNAs for replacement, and onco-miRNAs that are upregulated, which should be reduced using anti-miRNA [6,31]. Synthetic miRNAs are used to induce cell apoptosis and suppress tumor development [9]. Anti-miRNAs are chemically modified antisense nucleotides [31]. These antisense nucleotides demonstrated low toxicity and reduced immune response compared to plasmid DNA-based and protein-based drugs [32].

Mutation of miRNA expression have been found in most cancer types which originate from either genetic or epigenetic deficiencies [30]. The majority of miRNAs have demonstrated lower expression profiles in tumoral tissues than normal ones, acting as tumor suppressors [9,28,33]. Extrinsic factors such as the immune system and tumor stromal cells are also involved in the growth and spread of cancer by interacting with cancer cells and affecting their behavior. MiRNAs have a significant influence on each factor involved in cancer growth and dissemination, including chemotherapy resistance [28].

The two main challenges of miRNA-based therapy development are its stability and delivery [34]. The problems with miRNA delivery are the poor penetration of miRNAs into tumoral tissues and their quick degradation and clearance from the blood circulation [6,32]. Another barrier is the reticuloendothelial system (RES), which eliminates the oligonucleotides from the bloodstream [35]. Naked miRNAs show deficient intracellular delivery and also aggregation inside the endosomes, undergoing fast degradation or inactivation by nucleases that leads to a short half-life in the systemic circulation [32,36].

Nanoparticles improve the in vivo delivery of miRNAs and their tissue distribution, providing targeted delivery and limited adverse effects, finally leading to an enhanced therapeutic outcome [36]. The small size and low molecular weight make miRNAs valuable tools for formulating efficient delivery systems [32]. However, there are also challenges in miRNA delivery into target tissues because of their instability in biological fluids and tissues. Tissue-specific delivery, off-target effects, immunologic activation, and dosage determination are several difficulties in developing miRNA-targeted therapies [31]. The main issue with miRNA nanoparticles is the low transfection efficiency, which is due to low permeation through cell membranes as a result of their hydrophilicity, high molecular weight, and negative surface charge [28,36].

#### 2.2.2. siRNA

SiRNAs are products of the catalytic action of RNA replicase enzyme, first described and characterized in worms and plants. It has been proven that in plants, siRNAs are produced as a response to external stress or as a defense mechanism against viruses [24]. These sncRNAs can trigger more efficient and specific gene silencing than miRNA [37]. SiRNAs sparked interest in developing therapies that target pathologic RNAs [38]. Due to their inhibitory functions and tissue specificity in regulating specific target genes, siRNA showed significant potential and effectiveness in disease treatment [21]. SiRNAs show low stability in physiological fluids; therefore, they have a short half-life in the bloodstream and low permeability through biological membranes. These characteristics demand a proper carrier for its encapsulation and transport [38].

#### 2.2.3. piRNA

SncRNAs that interact with P-element-induced wimpy testis (PIWI) proteins are named piRNAs and represent single-stranded ncRNAs of 26–31 nucleotides. PiRNAs make up the largest class of ncRNAs, containing various nucleotide sequences [17,39]. PiRNAs play critical roles in gene regulation—more specifically, gene silencing during gene transcription and/or the post-transcription process—by binding to regulatory elements. Additionally, they have potential utility as biomarkers associated with cancer clinical features [39]. PIWI proteins possess an intrinsic binding affinity for piRNAs, and guide the sncRNAs to their targets and silence the transposon transcripts [24]. PiRNAs are also implicated in the silencing of retrotransposons at the post-transcriptional and epigenetic levels. They have increased stability due to the 5′-monophosphate and 2′-O-methyl groups in the 3′ terminal [17].

The mature piRNAs form a complex with PIWI proteins in the cytoplasm, which go back into the nucleus and fulfill their objective of blocking the transcription and maintaining genome integrity [17]. Even though there are still gaps regarding the biogenesis and function of piRNAs, research has managed to determine a possible differential expression of piRNAs in tumoral tissues compared with normal tissues and investigated their role in metastatic disease [17].

## 3. Types of Non-Viral Vectors

Compared with chemical drugs, nucleic acids are highly polar macromolecules that are unable to diffuse through cell membranes. Viral and non-viral delivery systems have been proposed as carriers for nucleic acids [40]. Viral vectors include retroviruses, adenoviruses, adeno-associated viruses, lentiviruses and herpes simplex viruses [5,41]. Retroviruses, adenoviruses, and lentiviruses have an approximate cargo capacity of 8 kilobases (1 kilobase = 37 kDa) [41,42]. Adeno-associated vector is one of the most common vectors used in gene therapy, accounting for 11 naturally occurring serotypes. The diversity of adeno-associated viruses alters their gene delivery characteristics, as there are more than 100 variants of adeno-associated viruses with different amino acid sequences that are successfully used in multiple gene therapy applications [41]. Non-viral vectors have a smaller cargo capacity even though sncRNAs have high molecular weights (i.e., approximately 13–16 kDa for the siRNA molecule) [37,42].

Viral vectors have the potential to deliver the desired genes to patients suffering from neurological disorders. They are used to transport nucleic acids to the brain parenchyma, proving a high transfection efficiency in the brain. Given that viruses cannot passively cross the blood-brain barrier, several administration routes have been developed to bypass the blood-brain barrier such as stereotaxic injection and injection into the cerebrospinal fluid [43]. Lentivirus, herpes simplex virus, adenovirus, and adeno-associated virus are the viral vectors that successfully achieved drug delivery into the brain [43]. As for genetic material delivery, adeno-associated viruses are currently the most advanced gene delivery vector for crossing the blood-brain barrier [44]. Non-viral vectors surfaces must be modified, either non-covalently with a coating or covalently by functionalization to reach their goal [45]. The lipophilic layers of lipidic nanoparticles facilitate the cargo passing across the brain membrane. Liposomal formulations provide features for passing the blood-brain barrier and releasing their content through endocytosis. Functionalization of liposomes surface with polyethylene glycol (PEG) or polysaccharides prevents their fast clearance [46]. Carbon nanotubes are promising nanocarriers for brain-specific therapies. These types of vectors also have surface functionalization, being able to bypass the blood-brain barrier [43]. An advantage of encapsulating the nucleic acids in nanoparticles is that the size of the carriers can be reduced, meaning that they can be easily designed to cross the blood-brain barrier [44,45].

The application of viral vectors is limited by immunogenic responses, inflammatory reactions, and the risks of inducing tumorigenic mutations [4,47]. Adeno-associated virus application is limited to a single administration because subsequent doses may cause severe immune reaction due to the immunogenicity [47,48]. Compared to viral vectors, non-viral vectors present reduced immunogenicity, as presented in Table 1. Viral vectors are associated with drawbacks such as repeated administration producing immune responses, mutagenesis, and carcinogenesis from nonspecific cell targeting [1,5]. Other limitations refer to problems encountered during the production of the viral vectors [4,40]. Therefore, non-viral vectors represent a more attractive alternative [5]. Non-viral delivery systems can achieve clinically relevant efficiency even though viral vectors demonstrated higher transfection efficacy and longer duration of target gene expression [32].

The main advantages of non-viral vectors over viral ones are the possibility of large-scale production, the reduced immune response and cytotoxicity, and the potential to be functionalized and targeted to specific sites [1,4,6]. Other strengths of non-viral approaches include ease of chemical characterization and reproducibility of production [11]. Even if the use of non-viral vectors has increased considerably, there is no available non-viral vector that achieves all the features of an ideal delivery system. However, the research in this field is under continuous improvement [4].

**Figure 2:**
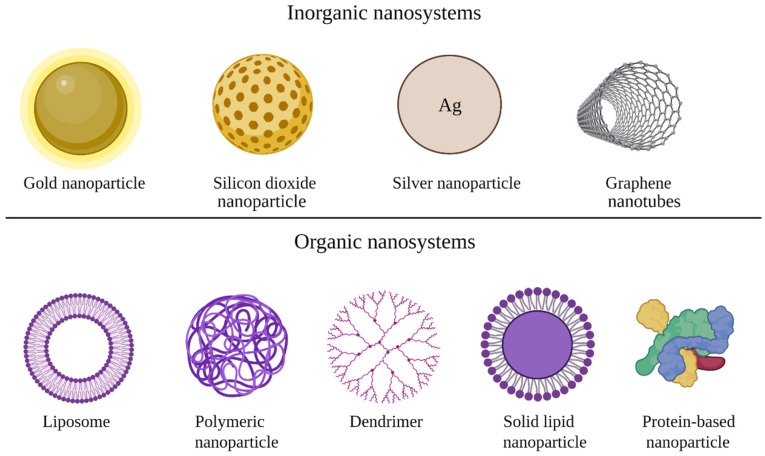
Types of nanosystems used for genetic material delivery. (Figure was created with BioRender.com (accessed on 13 June 2022)).

Nanosystems used as non-viral vectors are represented by inorganic nanoparticles (gold, silver, calcium phosphate, graphene, quantum dots, iron oxide, and silicon dioxide) and organic nanoparticles (liposomes, proteins/peptides, and polymer-based nanoparticles) [6,36]. The most studied types of nanoparticles used as non-viral vectors are illustrated in Figure 2. Additionally, Table 1 summarizes the advantages and limitations of some nanosystems used as non-viral vectors.

The most employed non-viral vectors in gene therapy are represented by cationic lipids and polymer-based systems that are chemically and physically stable, non-toxic, and non-immunogenic [1]. The nanoparticles used as non-viral delivery systems stabilize and protect the genetic material from nuclease degradation, increase their half-life in the bloodstream, and deliver the genetic material into the cytoplasm or nucleus, increasing potency [11,35]. The molecular strategies used for non-viral gene delivery in cancer treatment are numerous and complex: tumor suppressor gene replacement, oncogene silencing, miRNA targeted therapy, and genome editing [49].

Nanocarriers help miRNAs resist degradation by nucleases. The use of nanocarriers for oligonucleotide delivery also reduces the chances of immune rejection. Furthermore, nanoparticles have the advantage of surface modification by conjugation with different ligands, providing miRNAs with high specificity in delivery. They offer an enhanced cellular uptake of miRNA due to their small size and ability to link cell-penetrating peptides [6].

Nanoparticles encapsulating miRNA for cell-targeted delivery should possess colloidal stability to ensure the targeted delivery up to the desired site of action. Nanoparticles’ circulation time depends on their interaction with the biological environment, in some cases leading to their fast clearance [36]. It has been shown that the optimal size of nanoparticles for increased permeability and retention in tumor cells varies between 100 and 200 nm, also favorable to avoid spleen filtration and liver uptake [50]. Nanocarriers can prolong the circulation time of miRNA, allowing the accumulation in the tumoral tissue [51].

One of the nanoparticles’ particularities is their potential to passively accumulate in the tumor due to the enhanced permeability and retention effect. Nanoparticles can also release their content in a controlled manner, while the therapeutic efficacy may be further improved by accumulation at the tumor site through active targeting mechanisms. Moreover, nanoparticles are resistant to drug efflux mediated by multidrug resistance transporters, leading to drug accumulation at an effective intracellular concentration [52].

**Table 1:** Advantages and limitations of nanosystems used as non-viral vectors.

Nanosystem	Advantages	Limitations	Ref.
Silica nanoparticles	Biocompatibility Biodegradability High surface area Versatility Surface charge control Free dispersion throughout the body	Low transfection efficiency	[17,53]
Gold nanoparticles	Uniformity in size, shape, and biodistribution Tuned pharmacokinetics Increased surface area Biocompatibility Easy surface modification Controlled drug release Stability Strong gene carrying ability	Toxicity issues	[54,55,56]
Dendrimers	Presence of surface functional groups make them suitable for modification Good long-term stability	Expensive option	[9]
Polymeric nanoparticles	Biocompatible Biodegradable Non-immunogenic and non-toxic Easily fabricated in large quantities Low-cost Long-term stability	Prone to degradation Potential antigenicity Low transfection efficiency The need for surface modification	[9,57]
Liposomes	Biocompatibility Longer circulation time Amphiphilic	Expensive option Stability issues	[9,54]
Solid lipid nanoparticles	Biocompatibility Low toxicity Feasible to scale up Easy to sterilize	Low incorporation rates resulting from the crystalline structure of the solid lipid Lipid particle growth	[58,59]

### 3.1. Organic Nanoparticles

Polymeric nanoparticles are delivery vectors based on natural or synthetic polymers that show variation in their structure and molecular weight, which affect their physicochemical properties [6]. Polymeric nanoparticles proved the preservation of physicochemical properties such as zeta potential and particle size over long-term storage [9].

The potential of polymeric nanoparticles as delivery vectors stems from the large diversity of polymers [6]. Natural polymers such as chitosan, gelatin, sodium alginate or albumin, and the synthetic polymers such as polylactide (PLA), polyglycolide (PGA), and poly (lactide co-glycolide) (PLGA) are the most commonly used in polymeric nanoparticle preparation [60]. PLA and PLGA are biodegradable polyesters, approved by the FDA, and provide sustained delivery. Polymethacrylate is a vinyl-based polymer with limited transfection capacity due to its low ability to interact with membranes.

Cationic polymers-based systems for genetic material delivery are called polyplexes, and are more stable than lipidic nanoparticles. Polyplexes use natural polymers (e.g., chitosan) or synthetic polymers (e.g., polyethyleneimine) [4]. Cationic polymers are the most promising carriers for miRNA delivery, ensuring an increased cellular uptake. The polymers most used for miRNA delivery are PLGA or polyethyleneimine (PEI) [32]. The most used natural polymer for non-viral vector formulations is chitosan due to its lack of toxicity even at high concentrations [4].

Dendrimers are nanosized, polymeric structures consisting of a central core and two or more branches of different lengths, chemically synthesized using various polymers to obtain a variety of surface functionalities. They possess high drug loading capacity either by encapsulation or conjugation and have a high ability to deliver miRNA effectively [6,61]. Dendrimers have a terminal group that binds to the genetic material, and possess symmetrical shape [4]. Dendrimers for siRNA and miRNA delivery have been synthesized from polyamidoamine, polypropylenimine, carbosilane, and poly-L-lysine. One particular type of dendrimers which have been used for gene delivery is phosphorus dendrimers which contain a thiophosphate or cyclotriphosphazene core and thiophosphohydrazones extended branches [62]. Dendrimers possess great potential in drug and gene delivery, but their high cost limits their production and applicability. They are suitable for surface modification due to the presence of multiple functional groups. Generally, dendrimers show long-term stability [9]. Their toxicity profile is determined by the terminal amino group and the positive charge density [4].

The lipid-based nanoparticles used for nucleic acid delivery are liposomes, micelles, and solid lipid nanoparticles (SLNs) [63]. It has been demonstrated that lipid-based carriers are helpful for the systemic delivery of miRNAs, as they can protect miRNAs from degradation and increase their stability in the blood circulation. The formulation of nanocarriers with cationic lipids leads to the formation of electrostatic bonds between lipids and the negatively charged miRNAs, which provide the formation of cationic complexes, ensuring increased encapsulation of the miRNAs [32,47,63]. The structures formed by the interaction of the positively charged head group in lipids with negatively charged phosphate group in nucleic acids are named lipoplexes [4]. These structures can interact with negatively charged cell membranes, enhancing cellular uptake [63]. The major disadvantage of using cationic lipids for miRNA delivery is the cellular toxicity, since they can disturb the integrity of the cell membrane and reduce cell activity [36]. The use of neutral lipids that are less toxic can overcome this limitation, but the transfection efficacy of the obtained system is significantly decreased [32]. Either lipidic or neutral, the similarity of the lipidic nanoparticles’ composition to that of biological cell membranes promotes their interaction and cellular uptake [36].

SLNs contain lipids that are solid at room and body temperature [4]. Similar to other delivery systems, SLNs offer protection against nuclease degradation. SLNs with cationic lipids are used as miRNA carriers for cancer therapy; moreover, they are currently being investigated for siRNA delivery [4,32].

Liposomes are lipid-based bilayered nanoparticles containing an inner aqueous core, used to deliver chemotherapeutic drugs and nucleic acids into tumors [32,51]. They are considered the most effective nanosystems for nucleic acid delivery, with the following advantages: biocompatibility, biodegradability, high cellular transfection, controlled release, and structural flexibility [9,51,63]. Cationic liposomes are particularly suggested for nucleic acid delivery [63]. Additionally, using two types of lipids in their composition provides an increased encapsulation efficiency of the genetic material and stability of the liposomes [9]. Unilamellar liposomes encapsulate miRNA in the aqueous core, while multilamellar liposomes encapsulate miRNA between the lipid bilayers [36]. In recent years, research has shown that liposomes can considerably increase the efficacy of drug delivery [51]. As for disadvantages, liposomes show physical and chemical instability [9].

The main focus of the researchers in developing a proper drug delivery system is to obtain the desired characteristics, i.e., biodegradability, lack of toxicity, high drug loading, low cost, and facile preparation. Due to liposomes’ versatility in terms of their size, charge, and composition, they are the optimum carriers for siRNA incorporation and delivery [38].

### 3.2. Inorganic Nanoparticles

Silicon dioxide or silica nanoparticles are an attractive alternative for gene delivery due to the low toxicity of amino silicones used in their formulation. However, silica nanoparticles have a major disadvantage, namely, a decreased delivery efficiency in the presence of serum proteins [4].

Gold nanoparticles are inert and easy to prepare, and the possibility of surface modification with DNA can be exploited for cell transfection using the photothermal effect. This phenomenon induces thermal denaturation, resulting in gene release. Furthermore, the transfection efficiency of gold nanoparticles has proven to be comparable to that of lipidic nanoparticles but with lower in vitro toxicity. The major issue with gold nanoparticles is their chemical stability, leading to accumulation in cells [4]. Recently, studies proved that gold nanoparticles possess advantages for gene therapy, presenting improved transfection efficiency [56].

Carbon-based materials such as graphene oxide or carbon nanotubes are hexagonal networks of carbon atoms with cylindrical shape and a diameter of one nm [57,64]. Carbon nanotubes can be single-walled or multi-walled, each having different cellular uptake mechanisms. Single-walled carbon nanotubes have a localized effect, while multi-walled carbon nanotubes showed a prolonged effect in cells [54]. It has been shown that residual heavy metals induce cellular cytotoxicity, representing the biggest concern for the clinical use of carbon nanotubes [55]. Carbon nanotubes’ biological and chemical properties allow passive diffusion across the cell membrane or attachment to the cell surface with subsequent endocytosis, making them useful as delivery vehicles for various biomolecules [54,55,57]. Single-walled carbon nanotubes presented notable success in binding nucleic acids and significantly reduced tumor progression in melanoma [64].

## 4. Quality by Design (QbD) Approach in Non-Viral Vector Development

Pharmaceutical development aims to design a quality product, the manufacturing process of which can systematically deliver the product’s intended performance [65]. It should provide information regarding the material attributes and process parameters for understanding and enhancing the product quality and the manufacturing processes [65,66]. A systematic approach in pharmaceutical development should include the necessary tools for enhancing the quality of the product [66]. The QbD approach is defined by ICH Q8(R2) as “A systematic approach to development that begins with predefined objectives and emphasizes product and process understanding and process control, based on sound science and quality risk management” [65]. Pharmaceutical QbD has predefined objectives for formulation and manufacturing process development. This approach involves the use of specific tools, i.e., the Quality Target Product Profile (QTPP), Critical Quality Attributes (CQAs), Critical Material Attributes (CMA), and Critical Process Parameters (CPPs) [13,67,68]. This systematic approach contributes to robust formulation development and leads to a product of built-in quality. The factors that affect the product quality can be better understood using the Design of Experiment (DoE) tool [67]. To our knowledge, there have been no previous studies reporting the QbD-based development of non-viral vectors with application in gene therapy. In this sense, the present paper illustrates several procedures that help implement this approach for non-viral vectors for genetic material delivery.

Analytical Process Technology (PAT) is a QbD tool defined as “A system for designing, analyzing, and controlling manufacturing through measurements, during processing of critical quality and performance attributes of raw and in-process materials and processes, with the goal of ensuring final product quality” [14,69]. The identification of process variables that affect the quality attributes of the final product require PAT innovative sensors that are capable of real-time physical and chemical characterization and analysis. In order to fulfill the ICH and QbD requirements for manufacturing of robust nanoparticles formulations, real-time monitorization using PAT instruments is needed [70,71]. Currently, a PAT instrument is implemented for nanoemulations and nanosuspensions manufacturing for real-time size and size distribution monitorization [72]. This noninvasive and continuous measurement of nanoparticles’ physical characteristics reduces waste, minimizes batch rejects, and reduces production cycling time.

Data analysis is another important QbD tool that gives information about the relationship between the effect of CMAs and CPPs on the CQAs. The aim of this methodology is to study the multi-factorial relationship between the variables. The wide variety of real-time process analyzers used to monitor process parameters or material attributes generate a high number of complex data sets. The comprehension of statistical DoE, response surface methodology, optimization and multivariate data analysis is important for understanding the multi-factorial relationship between the variables [70,71,73]. Statistical methods such as regression analysis and Analysis of Variance (ANOVA) are the tools for data analysis [14].

There is no quality standard criteria or official methods proposed for the development of non-viral vectors for genetic material delivery. Given the difficulties in manufacturing and quality control that gene therapy requires, the QbD approach could be a useful tool in pharmaceutical development, especially for gene therapy products development. The QbD approach assists formulation performance in terms of composition (qualitative and quantitative) and process design, and helps understanding the sources of the variability. Moreover, it will assist in identifying critical formulations and process parameters that affect the product quality in order to determine the optimum formulation with a minimum number of experiments. Finally, this QbD approach can significantly benefit pharmaceutical industries involved in gene therapy by saving both time and cost. In this section we have proposed a quality profile, the critical quality attributes and monitoring methods for nanosystems used in gene therapy. The present paper proposes to bring together and to synthesize the existent information regarding the specific elements of QbD and gene therapy development based on the literature data, even though there are no published studies for nanosystems used in gene therapy for cancer therapy.

### 4.1. Quality Target Product Profile (QTPP) of Non-Viral Vectors

The QTPP comprises the quality characteristics that guarantee the efficacy and safety of the final pharmaceutical product, and represents the basis for the identification of the product CQAs [14,67]. Establishing the QTPP should be the first step in any pharmaceutical development process implementing QbD concepts [13]. The QTPP is set based on multiple efficacy and safety criteria, and must provide the route of administration, dosage form, dosage strength, drug release behavior, pharmacokinetic characteristics, quality criteria, shelf life, and container closure system [14,67,74]. An example of QTPP for non-viral vectors for gene therapy is provided in Table 2.

### 4.2. Critical Quality Attributes (CQAs) and Their Evaluation

#### 4.2.1. Critical Quality Attributes (CQAs)

CQAs are physical, chemical, biological, and microbiological characteristics identified based on the QTPP [13]. There are some limitations to identifying the CQAs of gene therapy vectors that involve previous expertise from the literature. Because many products remain in the early development stage, there is insufficient information regarding previous development of such products [74]. The use of risk assessment ensures a valuable identification of the CQAs. The CQAs need continuous monitoring to ensure that the defined variables do not affect the process performance [75]. As highlighted by previous publications in the field, we proposed the most important CQAs for non-viral vectors in Table 3. The CQAs listed in Table 3 are based on the results described in different studies for development of non-viral vectors for sncRNA delivery for cancer therapy.

Most studies considered as CQAs the particle size, the polydispersity index, and the zeta potential, but the reported values are different, so the information in Table 3 is only suggestive. Gan et al. prepared miRNA-124-loaded polymeric nanoparticles by the double emulsion method. The results obtained for the physicochemical characterization showed a mean particle size of 162 ± 1.25 nm, with a polydispersity index of 0.128 and a surface charge of 5.4 ± 1.45 mV [76]. Mohamed et al. also prepared miRNA-146a-loaded polymeric nanoparticles. The particle size was 244.8 ± 4.4 nm, with a zeta potential ranging between 5.9 mV and 11.1 mV [77]. Hong et al. reported the preparation of lipid-polymer hybrid nanoparticles loaded with afatinib and miR-139 for colorectal cancer treatment. The nanoparticles were prepared by the o/w emulsion method, and the morphology was determined by transmission electron microscopy. The encapsulation of afatinib and miR-139 in nanoparticles induced apoptosis and inhibited the migration and resistance of colorectal cancer cells [78]. Using a modified film dispersion method, Yan et al. prepared miRNA liposomes to treat triple-negative breast cancer by silencing the Slug gene. Human MDA-MB-231 cells were used to determine the cellular uptake of miRNA liposomes by flow cytometry and the intracellular localization by confocal microscopy. The liposomes showed an average particle size of 123.7 ± 0.1 nm, a polydispersity index of 0.252 ± 0.02, and a 6.4 ± 0.4 mV zeta potential. The cellular uptake was evaluated by the fluorescence intensity values and indicated a significant internalization of miRNA liposomes compared to control formulations. The intracellular localization showed that the miRNA liposomes were located in the cells’ mitochondria. Yan et al. also observed an inhibitory effect of miRNA liposomes on Slug gene and Slug protein expression and cancer cells growth [79]. Bochicchio et al. developed liposomes with phosphatidylcholine from egg yolk, cholesterol, and DOTAP, loaded with siRNA against the transcription factor E2F1 for colorectal adenocarcinoma treatment. The encapsulation efficiency was determined by a UV spectrophotometric assay, and was expressed as the percentage of siRNA encapsulated into the liposomes from the initial amount of siRNA. The particle size obtained was 24.86 ± 6 nm, and the encapsulation efficiency of siRNA was close to 100%. The siRNA-loaded liposomes demonstrated low cytotoxicity in cells compared to a commercial transfection agent. Furthermore, liposomes loaded with siE2F1 down-regulated the target in cultured colon adenocarcinoma cells and reduced cell growth [38]. Shi et al. prepared SLNs loaded with anti-miRNA-21 oligonucleotide for miRNA-based therapy in A549 human lung adenocarcinoma cell line. The study investigated the cellular uptake using a Confocal Laser Scanning Microscope and flow cytometry, and the cell viability was evaluated through the MTT assay. The characterization of SLNs revealed a mean particle size of 187.0 ± 2.6 nm and a mean zeta potential of 46.6 mV. As for the localization, SLNs were found in the cytosolic compartment and the nuclei. The intracellular uptake evaluated by flow cytometry was 74.6%. The MTT assay showed that anti-miRNA-21 SNLs inhibited cell proliferation at high concentrations compared to the control group [80]. Cui et al. analyzed the application of cationic liposomes incorporating miR-7 in ovarian cancer. The study evaluated the cellular uptake by fluorescence microscopy, the transfection efficiency and cytotoxicity in SKOV3 cells. The antitumor effect of the liposomes was evaluated after intravenous administration in mice over the course of three weeks, in comparison to free miR-7 and phosphate buffer solution. After the treatment, the mice were sacrificed, and the level of miR-7 was obtained using TaqMan miRNA assays. The miR-7 liposomes had a mean particle size of 127.43 ± 0.41 nm, a mean polydispersity index of 0.165 ± 0.004, and mean zeta potential of 9.23 ± 0.67 mV. The microscopy images obtained showed a high cellular uptake rate. Furthermore, regarding the transfection efficiency, 48 h after transfection with miR-7 liposomes, the expression of miR-7 increased by 58 times. The cytotoxicity study demonstrated that after 72 h incubation of miR-7 liposomes in SKOV3 cells, the viability of the cells decreased to 31%. In vivo experiments revealed that the miR-7 liposomes could inhibit the growth, invasion, and migration of ovarian cancer cells by inhibiting the expression of the epidermal growth factor receptor [81].

#### 4.2.2. Nanoparticle Physical Characterization Methods and Their Transfection Efficiency Determination

The physical properties of nanoparticles, such as particle size and surface charge, are essential for their cell internalization. These characteristics will also define the drug release kinetics, and their evaluation is required before testing the delivery system [82]. The most used characterization methods of nucleic acid-loaded nanoparticles are shown in Table 4.

The most commonly used techniques in molecular biology for determining the transfection efficiency include the luciferase assay and flow cytometry. The luciferase assay helps determine relative transfection performance, measuring luciferase activity, expressed as relative light units per milligram [83,84]. Using the principles of light scattering, light excitation, and emission of fluorochrome molecules to generate multiparameter data, flow cytometry is used as a method for assessing cellular uptake of nanoparticles [85]. Flow cytometry is a highly sensitive method for determining transfection efficiency, and can also be used for quantitative phenotyping of a multitude of cells [83].

**Table 4:** Characterization methods of nucleic acid-loaded nanoparticles.

Characteristics of Nanoparticles	Method	Principle	Ref.
Particle size	DLS	Measures particle size using Brownian motion	[79,86,87]
PDI			
Surface charge	Laser Doppler electrophoresis	Measures the particles’ frequency, obtaining electrophoretic mobility of the charged particles	[88]
Particle shape, morphology	Electron microscopy	Detection of reflected electrons, or transmission of electrons that pass through the sample	[82]
Cellular uptake	Flow cytometry	It uses fluorescence emission, which occurs as light from a laser beam strikes the moving particles. Based on the median fluorescence intensity, the area under the curve is calculated	[81,87,89,90]
	Cell lines fluorescence microscopy	After a predefined treatment, the cells are observed by fluorescence microscopy	
Intracellular localization	Confocal microscopy	The illumination and detection optics are focused on the same diffraction-limited spot in the sample, which is the only spot imaged by the detector during a confocal scan	[79,91]
Transfection efficiency	Cell lines RT-PCR	Nanoparticles are incubated with the cells and the level of nucleic acid is measured by RT-PCR	[79,81]
	Flow cytometry	The mean fluorescence intensity values correspond to the approximate number of fluorescent molecules associated with a cell	[83]
Cytotoxicity	Colony formation assay	Cells are treated after a predefined protocol, colored and the number of colonies is counted via an optical microscope	[81,92]
	Cell viability	Cells are treated after a predefined protocol with the MTT solution. The cell viability is expressed as the percentage of the absorbance of the sample to that of the untreated cells	
EE	Measurement followed by calculation	Determination of the percentage of genetic material encapsulated into non-viral vectors to the initial amount of genetic material included in the formulation	[38,79]

PDI—polydispersity index; DLS—Dynamic Light Scattering; RT-PCR—Real Time Polymerase Chain Reaction; MTT—Methyl Tetrazolium bromide; EE—encapsulation efficiency.

#### 4.2.3. Quantitative and Qualitative Evaluation of Encapsulated Genetic Material

The precise and sensitive miRNA detection is essential due to its valuable biomarker role. Real-time polymerase chain reaction (qRT-PCR) and microarray assays are the main techniques used for miRNA detection, and are susceptible to errors and need internal control. It is considered that nanotechnology could defeat the constraints of conventional quantification methods for sensitive miRNA detection [6]. Table 5 presents the most used quantitative detection methods for nucleic acids.

Nucleic acids separation is often carried out by electrophoresis, ultracentrifugation, or gel filtration [68,77,93,94,95]. Gel electrophoresis is also used for the determination of encapsulation efficiency of nucleic acids [87]. Usually, the encapsulation efficiency is calculated as the ratio of the initial amount of genetic material in the manufacturing of non-viral vector and the quantity measured in the non-viral vector [38,79]. The encapsulation efficiency of liposomes with nucleic acid usually ranges from 3 to 45% [63].

**Table 5:** Evaluation of encapsulated nucleic acids.

Objective	Method	Ref.
RNA or DNA quantification	UV-Vis spectrophotometry	[93,96,97]
	Fluorescence spectrophotometry	[79]
	Target-specific fluorescence detection	[93,96]
	Capillary electrophoresis separation of fluorescently labelled nucleic acids	[93,96]
	qRT-PCR	[97,98,99]
	Gel electrophoresis on 1% agarose gel	[87]
miRNA expression profiles	Provides 100% coverage of the miRNAs in the miRBase database	[96]

qRT-PCR—Real-time polymerase chain reaction.

### 4.3. Risk Assesment

Risk assessment represents an important step of the QbD approach that should be performed after CQA identification [74]. The risk management plan has the following steps: (1) risk identification, which involves the identification of the possible hazards and their consequences; (2) risk analysis, and risk evaluation that involve the estimation of the risk associated with the identified hazards and the comparison of the identified and analyzed risk against given risk criteria; and (3) risk control, which refers to the decision of acceptance, reduction, or elimination of risks [75,100]. Risk assessment aims to identify the CMAs and the CPPs that affect the quality of the formulation [68].

The two major quantitative risk assessment tools for the identification and evaluation of hazards are the Ishikawa diagram and failure mode and effects analysis (FMEA). In the Ishikawa fishbone diagram, also known as the cause-effect diagram, the CMAs and CPPs are assigned levels from low to high regarding their effect on the CQAs [68]. FMEA prioritizes the CPPs and CMAs by calculating a risk priority number (RPN) based on the evaluation of occurrence (O), severity (S), and detection (D) [14,101]. Another risk assessment method that can be applied in gene therapy is preliminary hazard analysis (PHA), a qualitative technique for identifying and ranking potential hazards [74,102]. Researchers propose the PHA-based CQA identification method in the gene therapy field due to the limited prior knowledge regarding product development [74].

### 4.4. Fabrication Process Understanding and Critical Process Parameters (CPPs)

The first step in a process improvement study is the identification of potential CPPs and their acceptable ranges [74,103]. CPPs are process parameters that must be monitored to ensure the desired CQAs [14]. They are categorized as high-impact CPPs, low-impact CPPs, or non-CPPs [103]. The CPPs are the parameters whose sensitivities significantly impact the QTPPs [104]. Table 6 summarizes the main preparation methods of nanoparticles for nucleic acid delivery and the process parameters considered critical for each method. Some of the presented CPPs have a more significant effect than others. The RPN calculates the severity of their impact on the quality of the intermediate of finished product. The CPPs of non-viral vector preparation must be controlled in order to ensure reproducibility [103]. For example, the mixing temperature and time of nanoparticles with the genetic material is an important CPP, and a multitude of studies indicate similar conditions for this parameter (incubation at room temperature for 30 min) [38,81,84,94,95]. Batch-to-batch reproducibility is another critical point for the scale-up, as well as the homogeneity of the product. A robust process should not be influenced by the process parameters variations [103]. For lipidic nanocarriers, the ethanol injection method is easier to scale-up compared to other preparation methods and leads to high stability vesicles [105]. As for viral vectors, scale-up involves challenges such as high costs and immunogenicity issues [44].

### 4.5. Excipients Used and Critical Material Attributes (CMAs)

CMAs include physical, chemical, biological or microbiological properties that must comply to ensure the desired CQAs [14]. CMAs greatly influence the outcome of the CQAs that are classified as high-risk factors [13]. The most recent studies focus on increasing the variety of new materials used for delivery systems. The materials used for genetic material delivery should be suitable for interaction with the human body, and their most important feature is their biodegradability [1].

Tumor-targeting ligands promote the accumulation in tumor tissues of nucleic acid nanocarriers. These ligands can be selected from antibodies or proteins (i.e., transferrin), peptides, aptamers, or small molecules (i.e., hyaluronic acid or folate). The surface of liposomes can be grafted with targeting fractions to achieve increased delivery and uptake into specific cell types [37,116]. Hyaluronic acid-conjugated nanoparticles ensure specific internalization, increased in vitro cytotoxicity, and targeted delivery of miRNA in breast cancer cells [35,36]. Deng et al. co-encapsulated negatively charged microRNA-34a with doxorubicin into hyaluronic acid and chitosan nanoparticles targeting hyaluronic acid receptors, which are overexpressed in breast cancer, for improved therapeutic effect. The resulting nanoformulation was a successful drug delivery strategy for enhanced anti-tumor therapy, proving synergistic tumor suppression effect [117].

There are hundreds of lipids developed for gene transfer with a positively charged hydrophilic head and a hydrophobic tail, and with a linker structure that connects both [4]. The hydrophilic headgroups can be sorted into the following categories: quaternary ammoniums, amines, and amino acids, or peptides, guanidiniums, and heterocyclic headgroups. These hydrophilic structures correlate with their transfection efficiency [47]. Each type of lipid provides different characteristics to the lipid/genetic material complex formed by the electrostatic bonds described above. Cationic lipids imitate the physicochemical proprieties of biological lipids, and the positive charge headgroup promotes the cellular uptake through electrostatic interactions with the negatively charged glycoproteins and proteoglycans of cells membranes [4,118]. However, the major obstacle in achieving therapeutic efficacy with nanoparticles exhibiting cationic surface charge is the rapid clearance by RES in the primary organs [5]. Cationic liposomes can cause cell changes, including cell shrinking, mitosis inhibition and cytoplasmatic vacuolization. Single-tailed cationic lipids are more toxic than double-tailed ones, resulting in decreased efficiency. Cationic phospholipids have more inhibitory capacity over protein kinase C, producing higher toxicity. The hydrophilic group from the cationic phospholipids structure determines the cytotoxic effect. The quaternary amine headgroup is more toxic than tertiary amine counterparts [119]. Moreover, the cationic lipids can interact with serum proteins which are also negatively charged. This interaction results in the formation of aggregates which afterward are eliminated by the liver and the spleen. Therefore, cationic lipids should be mixed with neutral lipids, also known as “helper lipids”, enhancing the stability and reducing the toxicity of lipidic nanoparticles [36].

Although the positive charge of lipids protects the genetic material from intracellular and extracellular nucleases, it also reduces the half-life of lipoplexes. PEG is used for surface protection of lipoplexes and for increasing their half-life and transfection efficiency [4,63]. PEG coating of liposomes provides longer circulation time in the blood, which leads to higher available concentrations of the nucleic acids at the targeted site, enhanced physical stability and improved tissue distribution [6,63]. Table 7 presents examples of phospholipids used for lipidic nanoparticles in gene delivery.

DOTMA was the first cationic lipid used for gene delivery and it is still used for non-viral vector delivery, ensuring successful entrapment of the genetic material, efficient delivery to cells, and significantly improving potential of non-viral agents for gene therapy [47,118]. After DOTMA, efforts have been made to improve the lipid formulation, which has led to the creation of DOTAP. DOTAP is a cationic phospholipid that demonstrated a higher efficiency for gene delivery when combined with a neutral lipid for cationic nanosystem formulation. The liposomal formulation of 100% DOTAP for gene delivery proved inefficient due to the positive charge density. DOSPA and DOGS are other cationic phospholipids that can act as a buffer due to their functionalized spermine head group [118].

The most used neutral helper lipids for gene delivery are DOPE and DOPC. Due to the inverted hexagonal packing structure, the neutral phospholipid DOPE presents higher transfection efficiency at low pH in comparison to DOPC [118]. DOPE and DOPC are amphiphilic molecules, which can form aggregates in both polar and nonpolar solvents at concentrations higher than their CMCs. Lipids oxidation can be affected by the lipid-water interfaces created during the aggregation [120].

CMC is a function of the number of hydrophobic carbon atoms in the lipid tail. Phospholipids with low CMC values show a detergent-like mechanism, forming fibers and increasing the bilayer thickness; therefore, the bilayers cannot form pores because of the incompatibility of protein-bilayer thickness. On the other hand, lipids with high CMC values form pores and do not exhibit a detergent-like behavior [121].

Studies that report the encapsulation of nucleic acids in lipidic non-viral vectors revealed that a phospholipid concentration of 20 mM is suitable for this purpose [122,123]. As reported by other authors, an increased phospholipids concentration leads to an increase in the liposome particle size [124]. Another important CMA that can affect CQAs is the genetic material:lipids ratio or genetic material:nanoparticles ratio, for which different values have been reported (i.e., 1:200, 1:250, 1:260, 1:700, 1:10) [77,78,79,87,125].

### 4.6. DoE and Linking CQAs to CMAs and CPPs

DoE is a set of statistical tools which include screening and optimization designs for determining the relationship between factors affecting a process and the output of the process [13,14]. The multivariate experiments carried out through DoE promote a better understanding of the impact of input variables alone and in combination with process parameters that affect the product [67]. The most important input factors are identified by systematic variation, leading to optimized output results [14]. DoE facilitates the evaluation of the impact of a large number of materials and process parameters on a biotechnological process [75]. This statistical tool also plays an important role in manufacturing process understanding [14,67]. The DoE’s advantages are providing better results with a minimum number of experiments, and evaluating CMAs and CPPs to obtain a product meeting the QTPP [13,14]. The use of experimental design provides the perfect strategy to develop and optimize a pharmaceutical product from a cost-effective point of view [126]. Screening DoE helps recognize input variables that are “insignificant” or “significant”, and is the most used type of design because it allows to evaluate a large number of CPPs and CMAs. Moreover, it saves time, effort, and materials [73,127].

## 5. Conclusions and Future Perspectives

Gene therapy is a field that predominantly develops in research laboratories that use gene transfer into the cell of a patient to treat or prevent a disease. Gene therapy is also known as the capacity for gene improvement by correcting mutated genes or site-specific modifications. It is important to know that more work is required to understand the advantages and disadvantages of each tool in gene therapy. In recent years, notable progress has been made in understanding ncRNAs. MiRNAs are the most studied small ncRNAs; it is now clear that miRNAs are potent gene regulators, although the understanding of their regulatory effects on transcription is limited. In addition, experimental techniques and model systems analysis should be employed when attempting to generalize miRNA capacities. More in vivo studies are required to determine whether miRNAs target specific cells under physiological conditions. Nevertheless, the current knowledge represents only a small fraction of the landscape of gene regulatory potential.

Nanotechnology is a field under continuous growth, and for that reason, progress on non-viral vectors for nucleic acid delivery is quickly acquired. The most important characteristics of nanosystems are zeta potential, particle size and size distribution, shape, morphology, cellular uptake, and transfection efficiency. The positively charged nanoparticles can encapsulate negatively charged genetic material by electrostatic interaction. Among all the nanosystems presented in this review, cationic liposomes and cationic polymers represent safe and efficient carriers for successful gene delivery.

QbD provides a systematic approach to drug development that intends to improve quality using analytical and risk-management methodologies, leading to reliable product development and manufacturing. Implementing this concept could bring a lot of advantages essential to developing non-viral vectors with reproducible physicochemical properties suitable for large-scale production. Regarding future perspectives, cationic lipids for gene delivery have become a major research tool for transferring genetic material into cells and there is great potential for progress in this direction.

## Figures and Tables

**Table 2:** Proposed QTPP for non-viral vectors for genetic material delivery.

Element	Target	Justification
Administration route	Intravenous	To improve the efficacy and bioavailability; direct availability in the bloodstream
Dosage form	Injection	Low volume production allows customisation to client/quantities
Delivery system element	Non-viral vector	Provides safer and more effective delivery of the genetic material
pH	7.35–7.45	To prevent or reduce vascular complications
Osmolarity	290–310 mOsm/L	To ensure tolerability
Particle size	Below 200 nm	To ensure penetration in the cell
Homogeneity	Monodisperse	To ensure system’s homogeneity
Enhanced therapeutic activity	High transfection efficiency (over 80%)	To improve system’s effectiveness
Storage condition	−60 °C ± 20 °C	To guarantee the stability of the genetic material
Improved safety	Lack of cytotoxicity, lack of haemolytic activity	To ensure appropriate biological requirements
Microbiological quality	Sterile and pyrogen-free	To avoid contamination with microorganisms; to ensure patient safety
In vitro release	Prolonged release	To ensure release according to a predefined release pattern, or to ensure spatio-temporal release of the payload

**Table 3:** Proposed CQAs for non-viral vectors.

CQA	Target	Is It Critical?	Justification
Particle size	100–400 nm	Yes	Internalization in tumor cells
PDI	0.1–0.5	Yes	Narrow size distribution; homogeneity of the nanosystem in terms of size
ZP	5–30 mV	Yes	Formation of electrostatic bonds between the vector and the cell environment
Surface modifications	Hyaluronic acid, transferrin, PEG	Yes	Decreased opsonization and phagocytosis; prolonged circulation
Cytotoxicity	High IC50	Yes	To ensure nanosystem safety
Cellular uptake	Efficient cellular uptake	Yes	To ensure penetration in the cell
Transfection efficiency	Over 80%	Yes	To ensure the desired biological effect

CQA—critical quality attribute; PDI—polydispersity index; ZP—zeta potential; PEG—polyethylene glycol.

**Table 6:** Preparation methods of nanoparticles for genetic material delivery.

Nanoparticle	Method	CPPs	Ref.
Gold nanoparticles	Layer-by-layer	Stirring speed and time	[8,106]
		Polyelectrolyte concentration	
	Laser ablation in liquid	Stirring speed and time	[107]
		Ultracentrifugation speed and time	
Liposomes	Film dispersion method	Incubation time, temperature	[79]
	Thin film hydration method	Evaporation time, pressure, temperature	[38,108,109,110,111,112,113,114,115]
		Hydration time, temperature	
	Ethanol injection method	Injection rate	[116]
Polymeric nanoparticles	o/w single emulsion method	Mixing speed, temperature	[77,78]
	Double-emulsion method	Sonication time, amplitude	[76]
		Stirring time, temperature	
SLN	Solvent diffusion method	Sonication time	[80,94]
		Agitation time, temperature, speed	
	Film-ultrasonic method	Sonication time	[95]

SLN—solid lipid nanoparticle; o/w—oil-in-water; CPPs—critical process parameters.

**Table 7:** Phospholipids used for lipidic nanoparticles manufacturing [36,118].

Type of Phospholipid		Name
Cationic	Monovalent	1,2-dioleoyl-3-trimethylammonium-propane (DOTAP)
		1,2-di-O-octadecenyl-3-trimethylammonium propane (DOTMA)
	Multivalent	Dioctadecylamidoglycylspermine (DOGS)
		2,3-dioleyloxy-N-[2(sperminecarboxamido) ethyl]-N,N-dimethyl-l-propanaminium trifluoroacetate (DOSPA)
Neutral		1,2-dioleoyl-sn-glycero-3-phosphoethanolamine (DOPE)
		1,2-dioleoyl-sn-glycero-3-phosphocholine (DOPC)
		Phosphatidylcholine

## Data Availability

Not applicable here.
